# Asthma self-assessment in a Medicaid population

**DOI:** 10.1186/1471-2458-9-244

**Published:** 2009-07-16

**Authors:** Ann C Wu, James Glauber, Charlene Gay, Tracy A Lieu

**Affiliations:** 1 Center for Child Health Care Studies, Department of Population Medicine, Harvard Medical School and Harvard Pilgrim Health Care, Boston, Massachusetts, USA; 2 Division of General Pediatrics, Children's Hospital Boston, Massachusetts, USA; 3 Neighborhood Health Plan, Boston, Massachusetts, USA; 4 Harvard Pilgrim Health Care, Boston, Massachusetts, USA

## Abstract

**Background:**

Self-assessment of symptoms by patients with chronic conditions is an important element of disease management. A recent study in a commercially-insured population found that patients who received automated telephone calls for asthma self-assessment felt they benefitted from the calls. Few studies have evaluated the effectiveness of disease self-assessment in Medicaid populations. The goals of this study were to: (1) assess the feasibility of asthma self-assessment in a population predominantly insured by Medicaid, (2) study whether adding a gift card incentive increased completion of the self-assessment survey, and (3) evaluate how the self-assessment affected processes and outcomes of care.

**Methods:**

We studied adults and children aged 4 years and older who were insured by a Medicaid-focused managed care organization (MCO) in a pre- and post-intervention study. During the pre-incentive period, patients with computerized utilization data that met specific criteria for problematic asthma control were mailed the Asthma Control Test (ACT), a self-assessment survey, and asked to return it to the MCO. During the intervention period, patients were offered a $20 gift card for returning the completed ACT to the MCO. To evaluate clinical outcomes, we used computerized claims data to assess the number of hospitalization visits and emergency department visits experienced in the 3 months after receiving the ACT. To evaluate whether the self-management intervention improved processes of care, we conducted telephone interviews with patients who returned or did not return the ACT by mail.

**Results:**

During the pre-incentive period, 1183 patients were identified as having problems with asthma control; 25 (2.0%) of these returned the ACT to the MCO. In contrast, during the incentive period, 1612 patients were identified as having problems with asthma control and 87 (5.4%) of these returned the ACT to the MCO (p < 0.0001). Of all 95 ACTs that were returned, 87% had a score of 19 or less, which suggested poor asthma control.

During the 3 months after they received the ACT, patients who completed it had similar numbers of outpatient visits, emergency department visits, and hospitalizations for asthma as patients who did not complete the ACT. We completed interviews with 95 patients, including 28 who had completed the ACT and 67 who had not. Based on an ACT administered at the time of the interview, patients who had previously returned the ACT to the MCO had asthma control similar to those who had not (mean scores of 14.2 vs. 14.6, p = 0.70). Patients had similar rates of contacting their providers within the past 2 months whether they had completed the mailed ACT or not (71% vs. 76%, p = 0.57).

**Conclusion:**

Mailing asthma self-assessment surveys to patients with poorly controlled asthma was not associated with better asthma-associated outcomes or processes of care in the Medicaid population studied. Adding a gift card incentive did not meaningfully increase response rates. Asthma disease management programs for Medicaid populations will most likely need to involve alternative strategies for engaging patients and their providers in managing their conditions.

## Background

Patient self-assessment is a key element of the Chronic Care Model [1]. Health care systems have successfully applied this intervention to improve the management of chronic diseases including diabetes [2-5] and hypertension [6-9].

Patient self-assessment also holds promise to improve care for asthma, an important chronic condition that affects more than 16 million adults and 7 million children in the United States [10]. Self-assessment has been found to predict and reduce future hospitalizations and emergency department (ED) visits among patients with asthma [8,11,12]. In addition, many patients with persistent asthma do not receive controller medications as recommended by evidence-based guidelines [13]. One important reason for underprescribing or underuse of controller medications may be that patients with asthma sometimes do not recognize that their asthma is poorly controlled [14,15]. Patient self-assessment surveys for asthma which alert patients that their asthma may not be adequately controlled may prompt patients to visit their asthma providers or to seek changes in their medication regimens. The Asthma Control Test (ACT), is a self-assessment survey that consists of five questions for adults and seven questions for children ages 4 to 11 years. The ACT is available at . The ACT has been found reliable, valid, responsive, and efficacious in identifying patients with suboptimal asthma control [16-18].

Although patient self-assessment for asthma has shown promise in privately insured populations, scant information exists about its effectiveness in Medicaid populations. Medicaid-insured and low-income persons are at higher risk for poor asthma control [19], and practical interventions that could improve the accuracy of asthma self-assessment and communication between patients and health care providers are especially needed.

The goals of this study were to (1) assess the feasibility of asthma self-assessment in a majority Medicaid population, (2) study whether adding a gift card incentive increased completion of the self-monitoring survey, and (3) evaluate how this self-assessment affected processes and outcomes of asthma care. We hypothesized that asthma self-monitoring in a Medicaid population would be feasible, that adding a gift card incentive would increase completion of the self-monitoring survey, and that self-monitoring would lead to processes of asthma care that result in fewer hospitalizations and ED visits.

## Methods

### Study Subjects

This study included adults and children aged 4 years and older insured by Neighborhood Health Plan (NHP), a community health center based Medicaid-focused managed care organization (MCO). NHP is contracted with MassHealth, the state Medicaid agency, to enroll members eligible for services, and MassHealth approved NHP's participation in the study. The study was also approved by the Human Studies Committee at Harvard Pilgrim Health Care. At the time of this study, the MCO insured more than 185,000 persons, including 125,000 who had Medicaid. Our study population consisted of patients with evidence of current problematic asthma control based on claims data. Criteria for inclusion in this population were developed by the medical director of the MCO with the goal of identifying patients who were certain to have problematic asthma control. Criteria included any one of the following: (1) high use of bronchodilators, defined as having at least one bronchodilator dispensing in the past 2 weeks and having had 3 or more dispensings of a bronchodilator in the past 4 months, (2) high use of systemic corticosteroids, defined as having at least one dispensing of a systemic corticosteroid in the past 2 weeks and having had 3 or more dispensings of a systemic in the past 4 months, or (3) an asthma-related ED visit within the past 6 weeks. As part of routine care, the MCO identified these patients with problematic asthma control and produced "asthma trigger reports." All trigger reports were distributed to the patients' primary care practice sites to alert providers that certain patients may have poorly controlled asthma. For a subset of patients, the MCO sent the patient's trigger report directly to the primary care provider with the GINA step chart for dose escalation [20].

### Study Design

**Figure 1:**
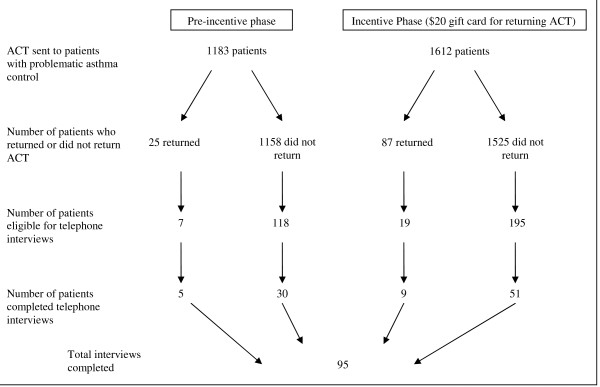
**Overall schematic of study**. During the pre-incentive phase, of 1183 patients with evidence of problematic asthma control and were sent mailings containing the ACT, 25 returned the ACT. During the incentive phase, 87 patients out of 1612 patients returned the ACT. During the pre-incentive phase, 125 patients were eligible for telephone interviews, and during the incentive phase, 214 patients were eligible. The main reason for ineligibility was lack of an accurate phone number of address. A total of 95 telephone interviews were completed.

This study used a pre- and post-intervention design to evaluate the intervention, self-assessment with incentives. The overall schematic for the study is depicted in Figure 1. In order to assess processes of care affected by our intervention, we interviewed a small proportion of the population studied in order to gain more detailed insight on the intervention.

### Intervention

This study involved two time periods: the pre-incentive and the incentive periods. During the pre-incentive (self-assessment without incentives) period, patients meeting the criteria for problematic asthma control were mailed the ACT with a cover letter from the MCO suggesting that it be completed in English or Spanish by each parent or guardian of each child ages 4 to 11 years or self-completed by children and adults ages 12 years and older. Responses used Likert-type rating scales and the sum yielded a score that suggested a range of control from poor to good. The cover letter informed the patients or parents that if the scores were 19 or less, their asthma was in poor control and they should contact their providers [18,21]. The cover letter also instructed patients to send the completed self-assessment survey back to the health insurance plan's asthma case manager, regardless of the score, in a return envelope with prepaid postage.

Concurrent with our intervention, asthma case managers at the MCO contacted select patients appearing on the trigger report. Patients were selected for outreach on the basis of having appeared on the report at high frequency, indicative of long-standing problematic asthma control, based on the clinical expertise of the asthma case managers. The asthma care management process did not change between the pre-incentive and incentive periods.

During the incentive period, all patients who were mailed the asthma self-assessment survey also were offered a $20 gift card to a local supermarket if the patient or parent returned the completed asthma self-assessment survey to the MCO.

### Claims Data

To evaluate clinical outcomes, we used computerized claims data to assess the number of hospitalizations and ED visits experienced in the 3 months after receiving the ACT. We also used computerized claims data to assess the number of fills of rescue and controller medications for asthma. We hypothesized that completion of the ACT would result in improved processes of care that would result in fewer hospitalizations and ED visits from asthma. In addition, we hypothesized that completion of the ACT would result in fewer numbers of fills of rescue medications and increased numbers of fills of controller medications.

### Telephone Interviews

We conducted telephone interviews with pre-incentive and incentive period patients to assess the effectiveness of the patient self-assessment strategy. For each patient, we conducted the follow-up interview within 8 weeks after the ACT was mailed in order to conduct the interview while the patients could remember receiving the mailing.

We conducted a second stage screening at the beginning of each telephone interview and excluded adults who reported (and children whose parents reported) that they had never been diagnosed with asthma. The structured telephone interview was conducted in English or Spanish and consisted of closed-ended questions. Parents who participated also received a $20 gift card for completing the telephone interview, independent of whether they had returned the ACT to the MCO. We attempted to conduct telephone interviews with all eligible patients.

During the pre-incentive phase, of 390 patients who were identified as having high asthma utilization, 262 were ineligible for our telephone interviews. Reasons for ineligibility were: we did not have an accurate phone number or address (n = 241); the family did not speak English (n = 10); the patient denied having asthma during the initial screen (n = 6); or a family member had previously enrolled in the study (n = 5). Of the 125 patients who were eligible for the study during the pre-incentive phase, 35 subjects completed the telephone interviews.

During the incentive phase, of 752 patients who were identified as having high asthma utilization, 538 were ineligible. Reasons for ineligibility were: we did not have an accurate phone number or address (n = 378); the patient or family member was already enrolled in the study (n = 113); the patient did not speak English (n = 35); the patient denied having asthma during the interview screen (n = 10), or the patient was unable to complete the survey due to cognitive or physical limitations (n = 2). Of the 214 patients who were eligible for the study during the incentive phase, 60 completed telephone interviews.

### Interview Instrument

The scripted telephone interview lasted approximately 20 minutes. After obtaining informed consent, questions were asked about baseline demographics including age, sex, race/ethnicity, highest grade completed in school, annual family household income, and whether the patient or parent was employed outside the home. We also assessed asthma medication use in the previous 12 months and administered the ACT.

In order to assess the processes of care potentially affected by the intervention, we interviewed a small subsample of our larger population. We assessed the frequency of patient contact with their asthma providers in response to information gleaned from the ACT. For those making contact, we assessed whether it was by telephone, in-person appointment, or whether the provider directly contacted the patient, or whether the provider asked the patient to make an appointment. For patients who had visits with their providers, we assessed whether the provider communicated that asthma control needed improvement, whether the provider recommended alternative or additional medications, discontinued a medication, or adjusted dosing of current medications.

In order to assess patient self-efficacy and perceived asthma control, our telephone interview included five questions that were adapted by RAND Corporation from a previous study [22]. We hypothesized that higher patient self-efficacy and perceived asthma control may be positively correlated with the likelihood of completing the ACT. These questions asked patients to report on a 5-point Likert scale whether their agreement with each statement (with 1 = strongly disagree to 5 = strongly agree) such as "If I do all the right things, I can successfully manage my asthma." We developed a summation score for self-efficacy and perceived control which ranged from 5 (high self-efficacy and perceived control) to 25 (low self-efficacy and perceived control).

We chose four questions on patient or parental expectations for symptoms (or asthma control) from a reliable and valid 8-item measure developed by Yoos et al [23]. Each question asked the parent to report on a 4-point Likert scale their agreement with the statement (with 1 = strongly agreed, 2 = agreed, 3 = disagreed, and 4 = strongly disagreed). The four questions on expectations for functioning with asthma had high inter-item correlation (Cronbach's alpha = 0.76) and were combined to form a summary measure with the possible scores ranging from 4 (lowest expectations) to 16 (highest expectations). We hypothesized that patients or parents who completed the ACT may have higher expectations for symptoms and asthma control. In addition, we hypothesized that among patients who completed the ACT, patients with higher expectations may experience the greatest decrease in hospitalizations and ED visits.

For patients who had visits with their asthma providers, we inquired whether the providers reported that their asthma control needed improvement, recommended medication changes, gave or reviewed a written asthma management plan, or suggested other interventions.

### Statistical Methods

We evaluated differences between the pre-incentive and incentive groups in rates of asthma outpatient visits, hospitalizations, ED visits, controller medications, and rescue medication using the t-test for continuous variables, Wilcoxon rank-sum test for non-parametrically distributed continuous variables or ordinal variables, and chi-square test for categorical variables. Analyses were conducted in R [24].

## Results

### Incentive Effect and Population Characteristics

During the pre-incentive period, 1183 patients were identified as having problems with asthma control; 25 (2.0%) returned the ACT to the MCO. During the incentive period, 1612 patients were identified and 87 (5.4%) returned the ACT to the MCO (p < 0.0001). Of all 112 ACTs returned, 87% had a score of 19 or less, consistent with poor asthma control. We completed telephone interviews with a total of 95 patients. Thirteen patients had returned the ACT to the MCO. In addition, 15 patients stated during their telephone interviews that they had completed the ACT but had not mailed it back to the health plan. Thus, a total of 28 patients had completed the ACT either based on receipt by the health plan or by self-report; we compared this group with the 67 patients who said they had not completed the ACT. In our analysis of telephone interview data, we grouped patients by whether they completed vs. did not complete the ACT, instead of returned vs. did not return the ACT. We felt that even if patients completed the ACT and forgot to mail the ACT to the MCO, completion of the ACT could result in changes in process and outcomes of care. In addition, it was possible that ACTs of patients could have been lost in the mail. Table 1 shows demographic and baseline clinical data from the 95 patients who participated (or whose parents participated) in telephone interviews. Based on these data, there were no significant differences between patients who did vs. did not complete the ACT to the MCO. Of the patients interviewed, 52% were white, 33% were black, and 11% were Latino. Seventy percent of families interviewed had a household income of $30,000 or less. There were also no significant differences for highest grade completed, household income, or types of medications used between the patients who completed the ACT and did not complete the ACT.

**Table 1:** Demographics of patients who completed the ACT versus did not complete the ACT

N = 95 n (%)	Completed ACT (n = 28)	Did not complete ACT (n = 67)	Total	p-value
Employed outside home?				0.11
Yes	8 (29%)	32 (48%)	40 (42%)	
No	20 (71%)	35 (52%)	55 (58%)	

Race/ethnicity				
White	15 (54%)	34 (51%)	49 (52%)	0.18
Black/African-American	8 (28%)	23 (34%)	31 (33%)	
Latino	2 (7%)	9 (13.5%)	11 (11%)	
Other	3 (11%)	1 (1.5%)	4 (4%)	

Highest grade completed				
Some high school or less	6 (21%)	12 (18%)	18 (19%)	0.21
High school graduate/some college	20 (72%)	40 (60%)	60 (60%)	
Post graduate/college	2 (7%)	15 (22%)	17 (22%)	

Household income				
$15,000 or less	11 (44%)	24 (37%)	35 (38%)	0.46
$15,000–$30,000	9 (36%)	20 (30%)	29 (32%)	
More than $30,000	5 (20%)	22 (33%)	27 (30%)	

Using this type of medication in the past 12 months				
Beta agonists	26 (38%)	62 (34%)	88 (35%)	0.60
Inhaled corticosteroid	14 (20%)	27 (15%)	41 (16%)	
Long acting beta agonists (LABA)	2 (3%)	4 (2%)	6 (2%)	
ICS/LABA	12 (17%)	38 (21%)	50 (20%)	
Oral corticosteroid	2 (3%)	16 (9%)	18 (7%)	
Mast cell stabilizers	9 (13%)	22 (12%)	31 (12%)	
Ipratroprium	4 (6%)	10 (5%)	14 (6%)	
Theophylline	0	4 (2%)	4 (2%)	

For our analyses, we compared patients who completed the ACT vs. patients who did not complete the ACT. We combined the patients who completed the ACT in the pre- and post-incentive phases in order to study our primary hypothesis that completion of the ACT would result in changes in processes and outcomes of asthma care. We hypothesized that adding a monetary incentive would increase completion of the ACT; however, we expected that completion of the ACT whether in the pre-incentive or incentive groups would result in similar changes in processes and outcomes of asthma care.

### Clinical Outcomes Based on Claims Data

Based on claims data analyzed for the 95 patients interviewed, 79% of patients who completed the ACT and 80% of patients who did not complete the ACT had 1 or more outpatient visits for asthma in the three months after receiving the ACT (p = 0.91). There were no differences in rates of outpatient visit, hospitalizations and ED visits in the 3 months after mailing ACT between the two groups (Table 2). Furthermore, patients who completed the ACT and did not complete the ACT had similar numbers of ED visits for asthma in the three months after the ACT was mailed. Similarly, no difference were seen in the number of prescription fills of beta_2_-agonists, inhaled corticosteroids, long acting beta_2_-agonists, or leukotriene inhibitors in the three months after the ACT was mailed. In addition, no differences were found in any of these outcomes between patients who were surveyed during the pre-incentive versus the incentive period.

**Table 2:** Outcomes stratified by completed ACT versus did not complete ACT

N(%)	Completed ACT	Did not complete ACT	p-value
Number of outpatient visits for asthma			0.91
0	6 (21%)	13 (20%)	
1	12 (43%)	27 (40%)	
2 or more	10 (36%)	27 (40%)	

Number of hospitalizations for asthma			0.77
0	25 (89%)	57 (85%)	
1	2 (7%)	5 (7.5%)	
2 or more	1 (4%)	5 (7.5%)	

Number of ED visits for asthma			0.28
0	19 (68%)	38 (57%)	
1	3 (11%)	17 (25%)	
2 or more	6 (21%)	12 (18%)	

### Processes of Care Based on Telephone Interviews

Completion of the ACT did not have an effect on whether the patient made contact with the provider, how the contact was made, and whether medication changes resulted from the contact (Table 3). As shown in Table 4, patients who completed the ACT had a slightly lower level of perceived control than those who did not complete the ACT (17.9 vs. 18.5, p = 0.23) and those who completed the ACT had lower expectations of asthma control (11.9 vs 12.6, p = 0.20). When comparing patients who were surveyed during the pre-incentive versus the incentive periods, no differences were noted for any of the questions on processes of care.

**Table 3:** Questions about contact with asthma providers, stratified by whether or not the patients completed the ACT

N = 95 n (%)	Completed ACT (n = 28)	Did not complete ACT (n = 67)	p-value
In past 2 months, how many times did you make contact with your provider?			
0	8 (29%)	16 (24%)	0.75
1	11 (39%)	32 (48%)	
2+	9 (32%)	19 (28%)	

If contact was made, contact was made by:			
Telephone	10 (59%)	25 (56%)	0.48
Appointment to see provider	6 (35%)	14 (32%)	
Provider called me	1 (6%)	1 (3%)	
Provider asked me to make appt	0	0	
Other	0	5 (8%)	

Provider felt asthma control needed improvement	12 (67%)	32 (71%)	0.97

Provider recommended another medication	7 (39%)	21 (46%)	0.83

Provider recommended discontinuing a medication	4 (22%)	10 (22%)	0.77

Provider started oral corticosteroid	4 (14%)	17 (25%)	0.36

Provider started inhaled corticosteroid	6 (21%)	17 (25%)	0.88

Provider gave or reviewed written treatment plan	10 (59%)	24 (55%)	0.99

Provider suggested other interventions	4 (22%)	19 (41%)	0.25

**Table 4:** Self-efficacy, perceived control, expectations for asthma, and attitudes towards providers, stratified by those who completed the ACT versus did not complete the ACT.

N = 95 n (%)	Completed ACT (n = 28)	Did not complete ACT (n = 67)	p-value
Mean perceived control score [SD]	17.93 [SD 2.11]	18.58 [SD 3.01]	0.23

Mean expectation score [SD]	11.92 [SD 2.02]	12.56 [SD 2.25]	0.20

How often did you see your asthma provider and not an assistant or partner?			
Never/rarely	7 (26%)	19 (29%)	0.37
Sometimes	11 (41%)	17 (26%)	
Often/always	9 (33%)	29 (45%)	

Not counting your doctor, how many different providers have given you asthma care in the past 12 months (mean [SD])	2.18 [SD 2.45]	2.65 [SD 3.86]	0.49

Did any providers call you to check on your asthma without your calling them first?			
Yes	11 (39%)	17 (26%)	0.22
No	17 (61%)	49 (74%)	

## Discussion

Our study has three key findings. First, mailing asthma self-assessment surveys to patients with poorly controlled asthma in a predominantly Medicaid-insured population showed no evidence of having improved their health care use and medication use patterns. Secondly, response rates to the self-assessment surveys were low, and adding a monetary incentive did not improve response rates in a way that would justify this intervention. Third, health plan administrative data can identify patients with current problematic asthma control.

In this study's population, mailing the ACT to patients with poorly controlled asthma was not associated with better outcomes or processes of care. A previous study found that self-assessment of asthma control with the Asthma Therapy Assessment Questionnaire (ATAQ) resulted in reduced numbers of hospitalizations and ED visits in a large urban medical group practice [11] and another study found that the findings of the ATAQ was highly correlated with the ACT score [25]; thus, we hypothesized that patients who completed the ACT would have fewer hospitalizations and ED visits as well because completion of the ACT would make patients aware of and take actions to rectify their poorly controlled asthma. To our knowledge, no published studies have demonstrated whether use of the ACT for asthma self-assessment affects any patient outcomes. Green and Foels report that they are studying this outcome but results are not yet available [26].

Our response rate was very low, most likely because we were attempting to outreach to a predominantly Medicaid population. Response rates to mailed surveys in the Medicaid population have been estimated to range 25–35% [27,28], but our response rates of 2–5.4% were still significantly lower. One explanation for our low response rate could be that our survey was targeted towards patients who had poorly controlled asthma; this sub-population may be particularly difficult to engage. Over 80% of patients identified on the asthma trigger report had previously been identified, and over a third had been identified >10 times. Such longstanding poor asthma control may signify or engender tolerance or resignation that requires more intensive outreach than a mailed survey. The low response rates may also signify reluctance by patients to send personal health information to an insurer. Adding the monetary incentive rate resulted in a higher response rate of 5.4% compared to 2.0% in the pre-incentive period (p < 0.0001), but this response rate is too low to warrant adoption of incentives in practice.

Our approach to identifying patients with poor asthma control based on computerized utilization data for medication fills and ED visits appears to be a valid method in a population of predominantly Medicaid enrollees. Of the patients who we were able to contact via our telephone survey, most had ACT scores suggesting poor asthma control. Thus, using computerized data has good potential to identify patients with poor asthma control for further intervention. Higher-intensity interventions, such as engaging their primary care physicians or case managers within the patient's practice, may be beneficial.

Despite the strengths of this study, a few caveats deserve mention. First, the response rate to our survey (28% during the pre-incentive and incentive phases) was relatively low. Nevertheless, we were able to conduct enough interviews for our objective of conducting a process evaluation of the intervention as a way to gain insight of potential barriers. Our goal was to identify patterns of experience rather than provide a systematic snapshot of the entire population. Furthermore, our power to identify an intervention effect on asthma-related health care use was limited, but no trends were identified. Furthermore, our utilization outcomes were limited to the three months after the ACTs were mailed; thus the follow up time period was relatively brief. Nevertheless, the medication fills were also similar whether patients completed or did not complete the ACT. We would expect that if completion of the ACT led to any changes in health care utilization, medication fills would change within three months of the mailings. As with other surveys, the ACT is prone to recall bias, however, studies have demonstrated the validity and reliability of this measure [25]. The seasonality of asthma may affect our results because of our pre-intervention and intervention design. The pre-incentive period occurred during July – October while the incentive period was during January – April. Nevertheless, similar numbers of asthma exacerbations and medication fills were noted in the pre-intervention and intervention periods. Although our study suggests that that ACT does not influence processes and outcomes of care, it is possible that our sample sizes were too small to detect a difference and that with more completed interviews, we could have detected an improvement in a process or outcome of care.

## Conclusion

Mailing asthma self-assessment surveys to patients with poorly controlled asthma yielded low completion rates and showed no evidence of improving asthma health services use or processes of care. Although health plans have the ability to reliably identify patients with problematic asthma control, additional interventions are needed to improve care for low income, multiethnic populations.

## Competing interests

The authors declare that they have no competing interests.

## Authors' contributions

ACW participated in the design and coordination of the study, supervised acquisition of data and statistical analysis, helped interpret the data, and drafted the manuscript. JW participated in the design and coordination of the study, helped with interpretation of the data, and helped to draft the manuscript. CG participated in the design and coordination of the study, participated in acquisition of data, and helped draft the manuscript. TAL participated in the design and coordination of the study, supervised the statistical analysis, participated in interpretation of the data, and helped draft the manuscript. All authors read and approved the final manuscript.

## Pre-publication history

The pre-publication history for this paper can be accessed here:



## References

[B1] Glasgow RE, Funnell MM, Bonomi AE, Davis C, Beckham V, Wagner EH (2002). Self-management aspects of the improving chronic illness care breakthrough series: implementation with diabetes and heart failure teams. Ann Behav Med.

[B2] Farmer A, Gibson O, Hayton P, Bryden K, Dudley C, Neil A, Tarassenko L (2005). A real-time, mobile phone-based telemedicine system to support young adults with type 1 diabetes. Inform Prim Care.

[B3] Guerci B, Drouin P, Grange V, Bougneres P, Fontaine P, Kerlan V, Passa P, Thivolet C, Vialettes B, Charbonnel B (2003). Self-monitoring of blood glucose significantly improves metabolic control in patients with type 2 diabetes mellitus: the Auto-Surveillance Intervention Active (ASIA) study. Diabetes Metab.

[B4] Johnson JA, Majumdar SR, Bowker SL, Toth EL, Edwards A (2006). Self-monitoring in Type 2 diabetes: a randomized trial of reimbursement policy. Diabet Med.

[B5] Piette JD, Weinberger M, McPhee SJ, Mah CA, Kraemer FB, Crapo LM (2000). Do automated calls with nurse follow-up improve self-care and glycemic control among vulnerable patients with diabetes?. Am J Med.

[B6] Bosworth HB, Olsen MK, Dudley T, Orr M, Neary A, Harrelson M, Adams M, Svetkey LP, Dolor RJ, Oddone EZ (2007). The Take Control of Your Blood pressure (TCYB) study: study design and methodology. Contemp Clin Trials.

[B7] Bosworth HB, Olsen MK, Neary A, Orr M, Grubber J, Svetkey L, Adams M, Oddone EZ (2008). Take Control of Your Blood Pressure (TCYB) study: a multifactorial tailored behavioral and educational intervention for achieving blood pressure control. Patient Educ Couns.

[B8] Peters D, Chen C, Markson LE, Allen-Ramey FC, Vollmer WM (2006). Using an asthma control questionnaire and administrative data to predict health-care utilization. Chest.

[B9] Rogers MA, Small D, Buchan DA, Butch CA, Stewart CM, Krenzer BE, Husovsky HL (2001). Home monitoring service improves mean arterial pressure in patients with essential hypertension. A randomized, controlled trial. Ann Intern Med.

[B10] Centers for Disease Control and Prevention. National Center for Health Statistics. http://www.cdc.gov/nchs/fastats/asthma.htm.

[B11] Patel PH, Welsh C, Foggs MB (2004). Improved asthma outcomes using a coordinated care approach in a large medical group. Dis Manag.

[B12] Schatz M, Zeiger RS, Mosen D, Vollmer WM (2008). Asthma-specific quality of life and subsequent asthma emergency hospital care. Am J Manag Care.

[B13] Donahue JG, Weiss ST, Livingston JM, Goetsch MA, Greineder DK, Platt R (1997). Inhaled steroids and the risk of hospitalization for asthma. JAMA.

[B14] Halterman JS, McConnochie KM, Conn KM, Yoos HL, Kaczorowski JM, Holzhauer RJ, Allan M, Szilagyi PG (2003). A potential pitfall in provider assessments of the quality of asthma control. Ambul Pediatr.

[B15] Fuhlbrigge AL, Guilbert T, Spahn J, Peden D, Davis K (2006). The influence of variation in type and pattern of symptoms on assessment in pediatric asthma. Pediatrics.

[B16] Schatz M, Mosen D, Kosinski M, Vollmer WM, O'Connor E, Cook EF, Zeiger RS (2007). Validation of the asthma impact survey, a brief asthma-specific quality of life tool. Qual Life Res.

[B17] Nathan RA, Sorkness CA, Kosinski M, Schatz M, Li JT, Marcus P, Murray JJ, Pendergraft TB (2004). Development of the asthma control test: a survey for assessing asthma control. J Allergy Clin Immunol.

[B18] Schatz M, Sorkness CA, Li JT, Marcus P, Murray JJ, Nathan RA, Kosinski M, Pendergraft TB, Jhingran P (2006). Asthma Control Test: reliability, validity, and responsiveness in patients not previously followed by asthma specialists. J Allergy Clin Immunol.

[B19] Smith LA, Bokhour B, Hohman KH, Miroshnik I, Kleinman KP, Cohn E, Cortes DE, Galbraith A, Rand C, Lieu TA (2008). Modifiable risk factors for suboptimal control and controller medication underuse among children with asthma. Pediatrics.

[B20] van Weel C, Bateman ED, Bousquet J, Reid J, Grouse L, Schermer T, Valovirta E, Zhong N (2008). Asthma management pocket reference 2008. Allergy.

[B21] Liu AH, Zeiger R, Sorkness C, Mahr T, Ostrom N, Burgess S, Rosenzweig JC, Manjunath R (2007). Development and cross-sectional validation of the Childhood Asthma Control Test. J Allergy Clin Immunol.

[B22] Katz PP, Yelin EH, Smith S, Blanc PD (1997). Perceived control of asthma: development and validation of a questionnaire. Am J Respir Crit Care Med.

[B23] Yoos HL, Kitzman H, McMullen A (2003). Barriers to anti-inflammatory medication use in childhood asthma. Ambul Pediatr.

[B24] R Development Core Team (2007). R: A language and environment for statistical computing.

[B25] Schatz M, Mosen DM, Kosinski M, Vollmer WM, Magid DJ, O'Connor E, Zeiger RS (2007). Validity of the Asthma Control Test completed at home. Am J Manag Care.

[B26] Green AW, Foels TJ (2007). Improving asthma management: one health plan's experience. Am J Manag Care.

[B27] Edlund C (1997). An effective methodology for surveying a Medicaid population: the 1996 Oregon Health Plan client satisfaction survey. J Ambul Care Manage.

[B28] Fredrickson DD, Jones TL, Molgaard CA, Carman CG, Schukman J, Dismuke SE, Ablah E (2005). Optimal design features for surveying low-income populations. J Health Care Poor Underserved.

